# Fabrication and Characterization of Electrospun Ocimum sanctum and Curcumin-Loaded Nanofiber Membrane for the Management of Periodontal Disease: An In Vitro Study

**DOI:** 10.7759/cureus.63678

**Published:** 2024-07-02

**Authors:** Poulami Chakraborty, Jaiganesh Ramamurthy

**Affiliations:** 1 Periodontics, Saveetha Dental College and Hospitals, Saveetha Institute of Medical and Technical Science, Chennai, IND

**Keywords:** tensile strength, sem, drug release pattern, membrane, nanofibers, electrospin, periodontitis, curcumin, ocimum sanctum

## Abstract

Background

Periodontal disease is a chronic inflammatory condition that gradually deteriorates the supportive tissues of teeth, eventually leading to tooth loss. Mechanical debridement stands as the gold standard method for treating periodontitis. However, antimicrobial therapy is recommended for optimal results when used alongside mechanical debridement. Numerous studies have investigated local drug delivery as an adjunct to mechanical debridement of affected tooth surfaces. Ocimum sanctum exhibits anti-inflammatory, antioxidant, and antimicrobial properties. Similarly, curcumin, as documented in the literature, demonstrates a broad spectrum of anti-inflammatory and antimicrobial effects. Electrospinning has demonstrated itself to be a highly effective method for fabricating drug-loaded fibers. Electrospun nanofibers containing Ocimum sanctum and curcumin are expected to exhibit greater efficacy due to their increased surface area, facilitating the dispersion of larger quantities of drugs, and their ability to control drug release when employed as a local drug delivery system. This study aims to fabricate and characterize the properties of nanofiber membranes loaded with Ocimum sanctum and curcumin using the electrospinning technique.

Methods

About 50 mg each of Ocimum sanctum and curcumin were blended with 15% polyvinyl alcohol and 2% chitosan polymer in a 4:1 ratio and left to stir overnight. A 10 mL syringe was filled with this solution, and an 18 G blunt-end needle charged at 15.9 kV was used for extrusion. Continuous fibers were collected onto a collector plate positioned 12 cm from the center of the needle tip, at a flow rate of 0.005 mL/min. The morphology of the fabricated membrane was assessed through scanning electron microscopy (SEM), the strength of the material was assessed through tensile strength analysis using INSTRON, an Electropuls E3000 Universal Testing Machine (INSTRON, Norwood, MA), and the drug release pattern was analyzed using Jasco V-730 UV-visible spectrophotometer (Jasco, Easton, MD).

Results

The morphology of this nanofiber showed a random distribution of fibers with no bead formation. The average diameter of the membrane was 383±102 nm, and the tensile strength of this material was 1.87 MPa. The drug release pattern showed an initial burst release of Ocimum sanctum, followed by a controlled release in subsequent hours. However, curcumin showed very little drug release because of its solubility.

Conclusion

In summary, the Ocimum sanctum and curcumin-loaded nanofibers exhibited robust tensile strength, a controlled drug release profile, and uniform drug distribution within the nanofiber membrane. Consequently, it can be concluded that curcumin nanofibers and electrospun Ocimum sanctum serve as valuable agents for local drug delivery in the treatment of periodontitis.

## Introduction

Periodontitis is a multifactorial chronic inflammatory disease that leads to the degradation of tooth-supporting tissues. It is characterized by the formation of periodontal pockets, bone loss, or gingival recession. Bacterial colonization of the tissues, resulting from the accumulation of calculus and plaque, triggers a localized inflammatory response by the host, leading to the destruction of tooth-supporting structures [1]. Gingivitis marks the initial stage of the disease, where the infection is confined to the gingival tissues, promoting the growth of various microorganisms, including *Tannerella forsythia, Porphyromonas gingivalis, Aggregatibacter actinomycetemcomitans, and Prevotella intermedia *[2].

Periodontitis is commonly treated with mechanical debridement of the affected root surface, to alter the environmental conditions of these microbiological environments and reduce the overall bacterial burden [3]. Additionally, mechanical debridement aids in establishing new attachments. However, the limitations of mechanical debridement in achieving an optimal root surface and providing accessibility for instrumentation in deeper pockets, coupled with the complexity of the microbial population, have spurred the search for supplementary therapeutic approaches aimed at decreasing the potential pocket depth for successful treatment of periodontal pockets [4].

According to the study by Van Winkelhoff et al. [4], the addition of extra antimicrobial medication could enhance clinical outcomes and might even be necessary for successful treatment. However, systemic antimicrobial therapy often raises concerns [5]. Over time, the use of synthetic drugs can lead to the development of resistant strains and other adverse effects. Consequently, controlled local delivery of antibacterial and anti-inflammatory medications was developed, which has shown superior efficacy compared to systemic administration methods for maintaining drug concentration in the periodontal pocket [6].

The emergence of drug resistance in pathogenic bacteria affecting humans and animals, coupled with the adverse effects of certain antibiotics, has sparked considerable interest in exploring novel plant-based antimicrobial alternatives. One primary advantage advocated for the therapeutic use of medicinal plants in treating various ailments is their safety, along with their affordability, potency, and accessibility [7]. Ocimum sanctum, renowned as the “Mother Medicine of Nature,” possesses properties that lower microbial count, oxidative stress, and inflammatory conditions [8]. Literature evidence indicates that Ocimum sanctum, as a local drug delivery agent, has demonstrated beneficial effects in managing periodontal diseases [9]. In addition to Ocimum sanctum, curcumin has exhibited numerous anti-inflammatory, antimicrobial, and antioxidant activities in the literature. Nagasri et al. [10] stated that curcumin, as an adjunct to scaling and root planing (SRP), yields better outcomes in periodontal parameters compared to SRP alone, and it has beneficial effects on managing periodontitis. Both herbal products prove effective for treating periodontitis when used as adjuncts to SRP. This study focuses on examining the combined effects of both Ocimum sanctum and curcumin as local drug delivery agents for managing periodontitis.

Various methods used for controlled drug delivery include oral irritants, gels, films, strips, fibers, and micro-particle systems. In recent years, electrospinning has garnered significant attention as a flexible and cost-effective technique for producing nanoscale fibers. Electrospinning is widely regarded as a straightforward method for creating polymeric nanofibers [11]. This method is based on the concept of electrohydrodynamics, which posits that an electric field causes polymeric threads with diameters ranging from 10 to 1,000 nm to be drawn [12]. During the electrospinning process, a polymer solution in a syringe with a capillary tip and a collector is subjected to high voltage. The collector gathers electrospun fibers into membranes. By controlling the voltage, concentration, and viscosity of the polymer solution, as well as the solvent composition, one can modify the properties of nanofibers [13]. Unlike other methods, the electrospinning technique enables researchers to control the rate at which fibers degrade, and consequently, the rate at which the drug is delivered [14]. On the other hand, administering a drug-loaded onto electrospun nanofibers to a patient is simpler compared to other methods. These nanofibers possess characteristics such as a large surface-to-volume ratio, a nanopore structure, and the ability to be chemically or physically modified [15]. This study aims to fabricate an electrospun nanofiber membrane loaded with Ocimum sanctum and curcumin, characterize this nanofiber membrane through scanning electron microscopy (SEM) analysis, evaluate its tensile strength, and analyze its drug release pattern.

## Materials and methods

The present study was conducted at the Saveetha Dental College and Hospital Research Lab. The study protocol received approval from the Institutional Review and Ethical Committee before commencement, under Reference No. SRB/SDC/PERIO-2204/23/154.

Fabrication of membrane

A 15% w/v solution of polyvinyl alcohol (PVA) was prepared by dissolving 15 g of PVA in 100 mL of distilled water and stirring at 700 RPM until a homogeneous mixture was formed. In addition to PVA, a 2% chitosan solution was prepared by adding 1 g of chitosan to 50 mL of acetic acid solution, which was stirred overnight to achieve homogeneity.

**Figure 1:**
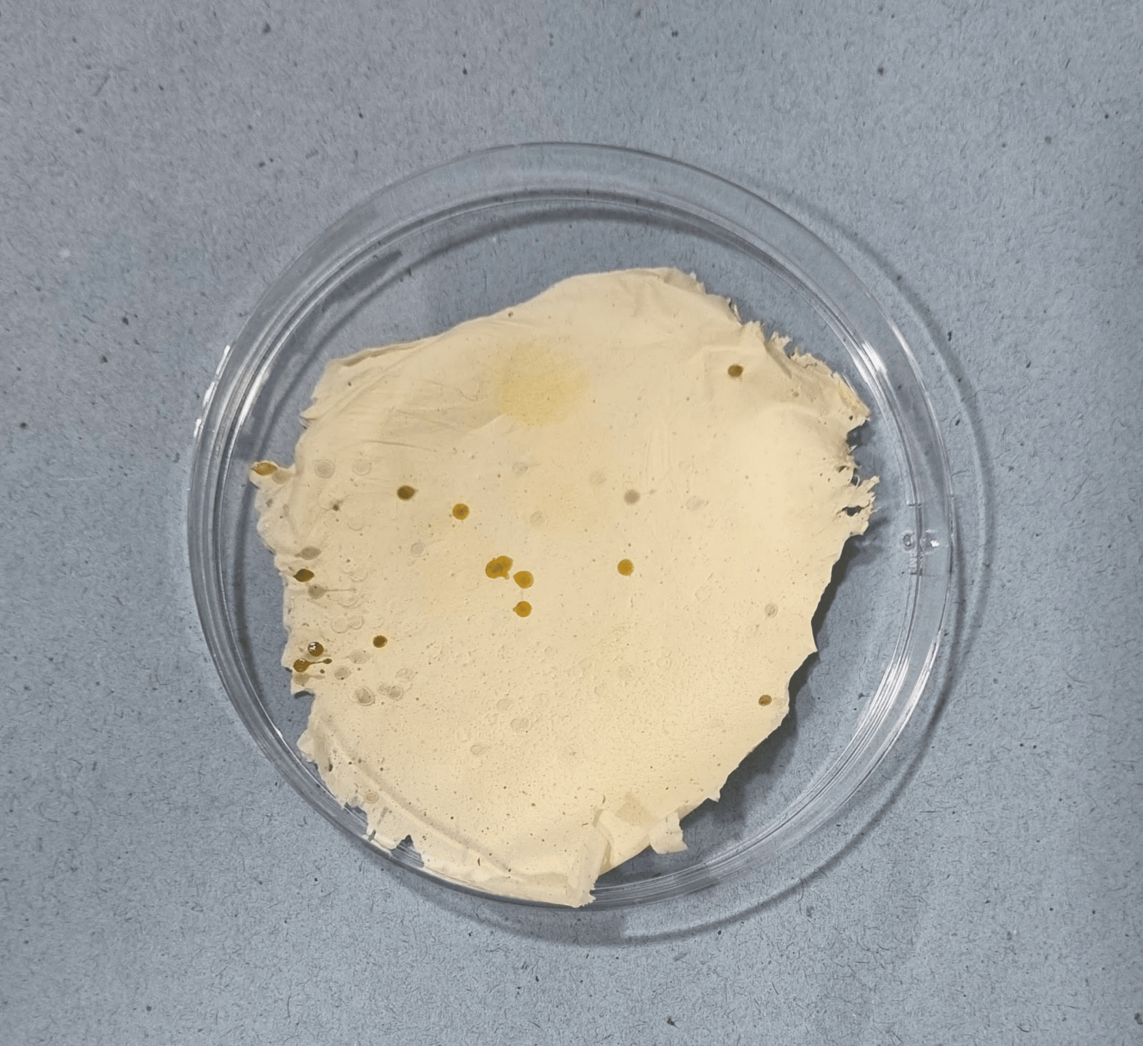
Fabricated Ocimum sanctum and curcumin-loaded nanofiber membrane

A mixture consisting of 15% w/v PVA and 2% chitosan in a 4:1 ratio was prepared, along with 50 mg each of Ocimum sanctum and curcumin. The solution was stirred homogeneously for 24 hours. Subsequently, the polymer solution was loaded into a 10 mL syringe, and extrusion was performed using an 18 G blunt-end needle charged at 15.9 kV. Continuous fibers were collected onto a collector plate positioned 12 centimeters from the center of the needle tip at a flow rate of 0.005 milliliters per minute. The fabricated fibers underwent further analysis, as depicted in Figure 1.

Scanning electron microscopic analysis

Visualizing and assessing the surface texture, size, shape, and other attributes of the nanofiber membrane were achieved through SEM analysis. This method enables both quantitative and qualitative assessment of the sample's surface composition. With SEM, it is possible to quantify the size of pores and analyze them using imageJ software (National Institutes of Health, Bethesda, Maryland). The sample was first coated with platinum using a sputter coater at room temperature. Following platinum plating on a stub (JSM-IT800, JEOL, Germany), the overall morphology of the membrane samples was assessed using SEM. Images were captured at different magnifications during the process, which was carried out at a 5 kV acceleration voltage.

Tensile strength analysis

This test employed a coaxial load to assess the strength of the material. The experiment used to determine the mechanical strength of a material applies a gradual force using static electricity. The tensile strength of the fabricated membrane was assessed using INSTRON, an Electropuls E3000 Universal Testing Machine (UTM) (INSTRON, Norwood, MA). Megapascals were the unit of measurement for tensile strength. To evaluate the membranes' tensile strength, they were cut into rectangular strips (30 mm x 5 mm). A speed of 1 mm per minute was chosen for the crosshead. The arms of the universal testing device were fitted with the membranes. There was no moisture present when the tensile strength was assessed. To obtain the values of tensile strength, each sample was stretched.

Analyzing the drug release pattern

To analyze the drug release pattern from the membrane, a 1 cm x 1 cm section of the fabricated membrane was cut and immersed in 1 mL of phosphate buffer solution (PBS). Subsequently, the solution was collected and analyzed under Jasco V-730 ultraviolet (UV)-visible spectroscopy (Jasco, Easton, MD) at various time intervals, namely one hour, three hours, six hours, 12 hours, 24 hours, and 48 hours. UV-visible absorption spectra of the ligand and the metal complex were examined within the wavelength range of 200-1,100 nm by dispersing 1 mL of the collected samples at different time intervals using a UV-visible spectrophotometer.

## Results

Scanning electron microscopic analysis

**Figure 2:**
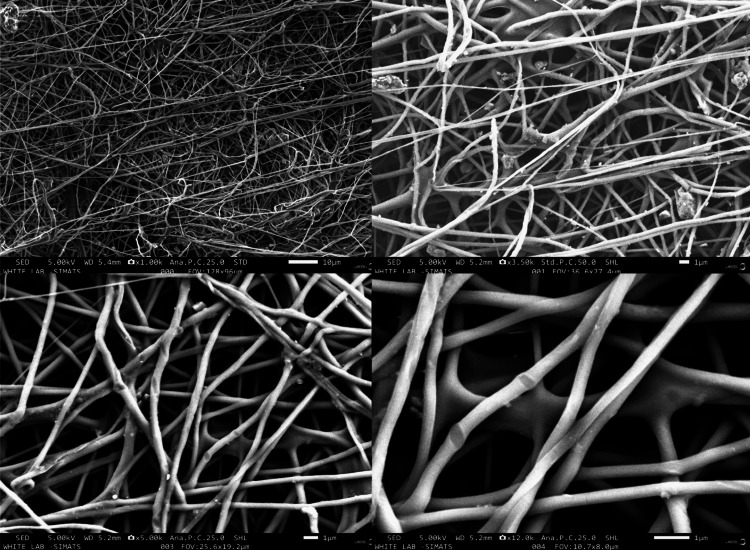
Scanning electron microscopic images of Ocimum sanctum and curcumin-loaded nanofiber membrane of various magnifications x1.00k, x3.50k, x5.00k, x12.0k at 5.00kV

The morphological appearance of this electrospun membrane was analyzed through SEM. The micrograph images are presented in Figure 2, illustrating continuous, long nanofibers randomly distributed across the fibrous membrane, with no visible accumulation of polymer mass in the form of beads. The average diameter of the fibers in the membrane containing Ocimum sanctum and curcumin was found to be 383 ± 102 nm. Ocimum sanctum and curcumin are uniformly dispersed throughout the polymer and membrane, as evidenced by the absence of bead formation and the apparently even distribution of nanofibers within the nanofiber cluster.

Tensile strength analysis

**Figure 3:**
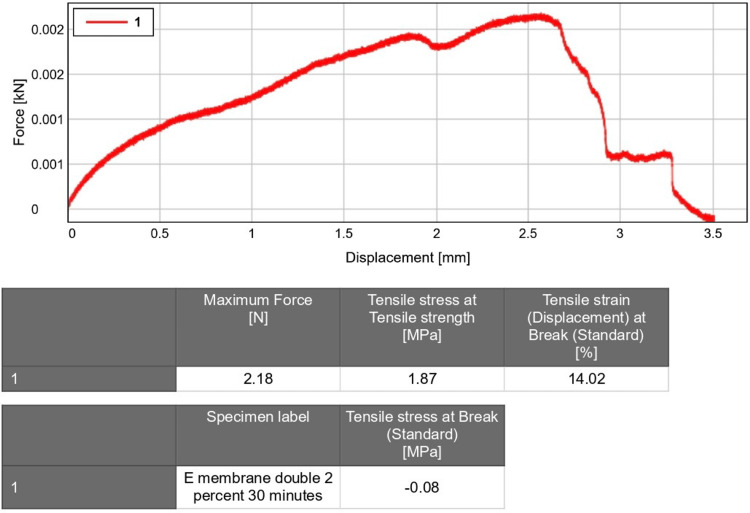
Tensile strength of Ocimum sanctum and curcumin-loaded nanofiber membrane

The tensile strength test was conducted using the UTM, which involves applying a controlled force to the samples and measuring the resulting deformation until the samples rupture. The maximum force applied was 2.18 N, and the tensile strain at break was 14.02%. The obtained tensile strength value was 1.87 MPa (Figure 3).

Analyzing the drug release pattern

**Figure 4:**
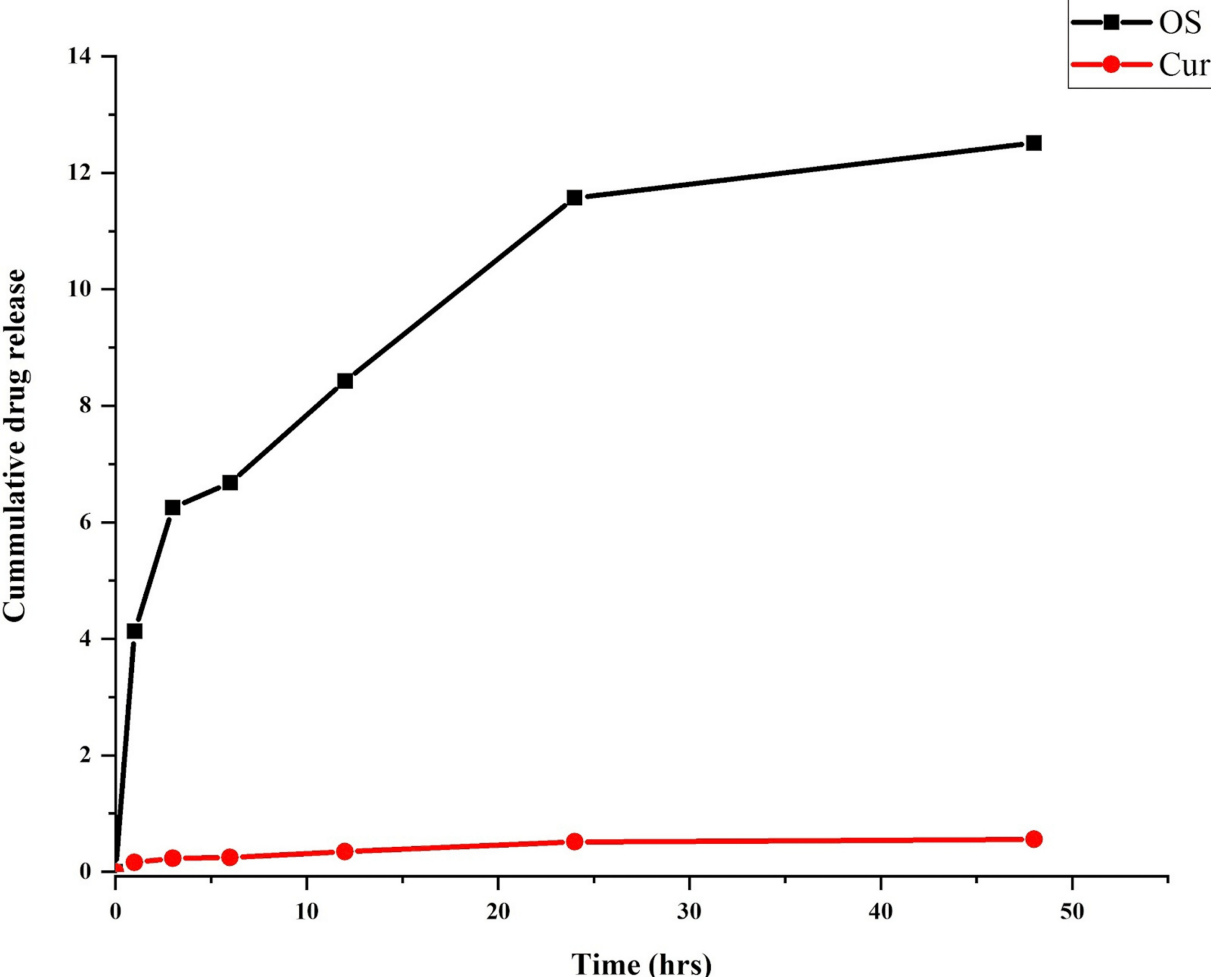
Drug release pattern at different time intervals of Ocimum sanctum and curcumin-loaded nanofiber membrane

The in vitro release behavior of Ocimum sanctum and curcumin-loaded nanofibers is illustrated in Figure 4. It was observed that the release patterns of both drugs differed. There was an initial burst release of Ocimum sanctum within the first four hours, followed by a controlled release in the subsequent hours. However, curcumin exhibited distinct behavior; its release rate was very low, attributable to its solubility nature.

## Discussion

Recently, there has been considerable interest in researching local drug delivery for the treatment of periodontitis. Local drug delivery in periodontitis entails a targeted therapeutic approach aimed at delivering antimicrobial agents, anti-inflammatory drugs, or other bioactive compounds directly to the periodontal pockets to treat and manage the disease [16]. This method offers several advantages over systemic administration, including increased drug concentration at the site of infection, reduced systemic side effects, and enhanced patient compliance. According to Chinna et al.’s [17] study, for severe types of periodontal diseases, the combination of SRP with tetracycline-loaded local drug delivery system (LDDS) fibers serves as a suitable alternative. In addition to clinical considerations, the kinetics of the LDDS were evaluated in vitro, revealing that this particular fiber type was effective against* P. gingivalis* in a simulated periodontal disease environment and delivered a controlled amount of medication for a therapeutic period of 10 days [18]. While the local use of tetracycline in periodontal therapy may reduce bacterial infection, it may not be optimized for other therapeutic purposes such as anti-inflammatory, antioxidant, and regenerative effects. The use of natural compounds represents another therapeutic approach against periodontitis that has garnered significant attention in recent years [19].

Ocimum sanctum and curcumin are renowned for their diverse pharmacological properties, including anti-inflammatory, antioxidant, antimicrobial, and anticancer activities. However, their clinical utility is often hindered by poor aqueous solubility, low bioavailability, and rapid metabolism. Encapsulating these bioactive compounds within electrospun nanofibers enhances their stability, allowing for sustained release and prolonged therapeutic effects. Moreover, the synergistic interactions between Ocimum sanctum and curcumin may potentiate their therapeutic efficacy, leading to improved outcomes in various disease conditions [9,10].

Numerous studies are being conducted to investigate the potential of electrospun nanofibers as a localized medication delivery mechanism [20]. In terms of adjusting or fine-tuning the releasing characteristics of a drug, electrospinning is the most adaptable, programmable, and excellent method for creating nanofibers. Excellent control and command over the shape, orientation, size, and dimension of nanofibers, as well as a variety of encapsulation techniques, render it the best [21]. Chitosan and PVA were the polymers used in multiple investigations to create membranes; these were selected primarily because they provide mechanical stability, are resorbable, and have been approved for use in medicine [22,23]. According to the study by Paikpitak et al. [24], the weight ratio of PVA to CS at 80:20 in 2% acetic acid has been incorporated into the present methodology to form a uniform fiber structure.

In this study, nanofiber membranes loaded with Ocimum sanctum and curcumin were fabricated using the electrospinning technique. Subsequently, a 10mm x 10mm section of the membrane was excised to investigate the drug release profile using a v-730 UV-visible spectrophotometer. The morphology of the membrane was assessed via SEM, revealing randomly oriented fibers without bead formation, consistent with prior research [24, 25]. The average diameter of the fibers was measured to be 383 ± 102 nm. Additionally, the membrane exhibited a tensile strength of 1.87 MPa, indicating favorable mechanical properties.

In a study led by Wsoo et al. [21], electrospinning was utilized to create PCL nanofibrous membranes and cellulose acetate nanofibrous membranes loaded with various concentrations of vitamin D3. These nanofibers exhibited narrow distribution, smooth morphology, and a gradual drug release rate, which is essential for achieving long-term controlled drug release. Similarly, Jiang et al. [26] concluded in their study that the drug release behavior can be modulated by adjusting the diameter of the nanofibers. Additionally, Hu et al. [27] successfully produced electrospun PLGA/GE nanofibers loaded with both 5-FU and cefradine using emulsion electrospinning. These fibers possessed favorable hydrophilic properties and mechanical strength, with drug release patterns characterized by initial burst release followed by gradual release. Moreover, Awan et al. [28] fabricated a curcumin-loaded antimicrobial nanofibrous membrane via electrospinning and assessed its morphology and chemistry through SEM and Fourier transform infrared (FTIR) analysis. Their findings indicated successful curcumin absorption into the nanofibrous mat, resulting in consistent thickness and morphology of the generated nanofibers. These findings align with the results of our own investigation.

Furthermore, Rahalkar et al.'s [29] study showed that both tulsi and curcumin, individually used as adjuncts to SRP, are effective agents in reducing plaque index, gingival index, and clinical attachment gain when compared to SRP alone. Another study by Agrawal et al. [30] claimed that while curcumin has a greater effect on reducing plaque, tulsi extract is more successful at eliminating red-complex bacteria. Taking this into consideration, our study was designed to create a locally deliverable component and derive the combined benefits of curcumin and Ocimum sanctum. The resulting nanofiber membrane exhibits favorable tensile strength and controlled release properties.

Limitations

This study suggests an advanced drug delivery system for the management of periodontitis. However, this study does have certain limitations. Specifically, the swelling and degradation rates were not evaluated in the in vitro investigation. Consequently, to validate the conclusions of the study, in vivo studies and randomized controlled trials involving larger populations are warranted.

## Conclusions

The findings of the research suggest that a smooth nanofiber membrane can be formed by combining curcumin with the natural herb Ocimum sanctum through the electrospinning method. Additionally, the research highlights the potential of combining curcumin and Ocimum sanctum in a nanofiber membrane for local drug delivery in treating periodontal disease. This approach presents a promising avenue for advancing drug delivery systems, offering the combined benefits of both herbs in addressing the complexities of periodontitis.

## References

[1] Haffajee AD, Socransky SS (1986). Attachment level changes in destructive periodontal diseases. J Clin Periodontol.

[2] Ding J, Zhao C, Gao L (2023). Metabolism of periodontal pathobionts: their regulatory roles in the dysbiotic microbiota. Mol Oral Microbiol.

[3] Greenstein G (1992). Periodontal response to mechanical non-surgical therapy: a review. J Periodontol.

[4] Winkel EG, van Winkelhoff AJ, Barendregt DS, van der Weijden GA, Timmerman MF, van der Velden U (1999). Clinical and microbiological effects of initial periodontal therapy in conjunction with amoxicillin and clavulanic acid in patients with adult periodontitis. A randomised double-blind, placebo-controlled study. J Clin Periodontol.

[5] Krayer JW, Leite RS, Kirkwood KL (2010). Non-surgical chemotherapeutic treatment strategies for the management of periodontal diseases. Dent Clin North Am.

[6] H R R, Dhamecha D, Jagwani S (2019). Local drug delivery systems in the management of periodontitis: a scientific review. J Control Release.

[7] Ashraf MV, Pant S, Khan MA (2023). Phytochemicals as antimicrobials: prospecting Himalayan medicinal plants as source of alternate medicine to combat antimicrobial resistance. Pharmaceuticals (Basel).

[8] Pattanayak P, Behera P, Das D, Panda SK (2010). Ocimum sanctum Linn. A reservoir plant for therapeutic applications: an overview. Pharmacogn Rev.

[9] Deepika BA, Ramamurthy J (2023). Effect of Ocimum sanctum L as LDD in periodontal therapy. Bioinformation.

[10] Nagasri M, Madhulatha M, Musalaiah SV, Kumar PA, Krishna CH, Kumar PM (2015). Efficacy of curcumin as an adjunct to scaling and root planning in chronic periodontitis patients: a clinical and microbiological study. J Pharm Bioallied Sci.

[11] Garg T, Goyal AK (2014). Biomaterial-based scaffolds--current status and future directions. Expert Opin Drug Deliv.

[12] Pankongadisak P, Sangklin S, Chuysinuan P, Suwantong O, Supaphol P (2019). The use of electrospun curcumin-loaded poly (L-lactic acid) fiber mats as wound dressing materials. J Drug Deliv Sci Technol.

[13] Khil MS, Cha DI, Kim HY, Kim IS, Bhattarai N (2003). Electrospun nanofibrous polyurethane membrane as wound dressing. J Biomed Mater Res B Appl Biomater.

[14] Torres-Martinez EJ, Bravo JMC, Medina AS, González GLP, Gómez LJV (2018). A summary of electrospun nanofibers as a drug delivery system: Drugs loaded and biopolymers used as matrices. Curr Drug Deliv.

[15] Sun XZ, Williams GR, Hou XX, Zhu LM (2013). Electrospun curcumin-loaded fibers with potential biomedical applications. Carbohydr Polym.

[16] Wei Y, Deng Y, Ma S (2021). Local drug delivery systems as therapeutic strategies against periodontitis: a systematic review. J Control Release.

[17] Chhina S, Rathore AS, Juneja S (2015). Alpha-2-macroglobulin levels in gingival crevicular fluid pre- and post-scaling and root planing with adjunctive tetracycline fibers in chronic periodontitis: a randomized controlled trial. J Contemp Dent Pract.

[18] Vijayalashmi R, Ravindranath SM, Jayakumar ND, Padmalatha Padmalatha, Vargheese SH, Kumaraswamy KL (2013). Kinetics of drug release from a biodegradable local drug delivery system and its effect on Porphyromonas gingivalis isolates: an in vitro study. J Indian Soc Periodontol.

[19] Viglianisi G, Santonocito S, Lupi SM, Amato M, Spagnuolo G, Pesce P, Isola G (2023). Impact of local drug delivery and natural agents as new target strategies against periodontitis: new challenges for personalized therapeutic approach. Ther Adv Chronic Dis.

[20] Son YJ, Kim WJ, Yoo HS (2014). Therapeutic applications of electrospun nanofibers for drug delivery systems. Arch Pharm Res.

[21] Wsoo MA, Razak SI, Bohari SP, Shahir S, Salihu R, Kadir MR, Nayan NH (2021). Vitamin D(3)-loaded electrospun cellulose acetate/polycaprolactone nanofibers: characterization, in-vitro drug release and cytotoxicity studies. Int J Biol Macromol.

[22] Homayoni H, Ravandi SA, Valizadeh M (2009). Electrospinning of chitosan nanofibers: processing optimization. Carbohydrate Polymers.

[23] Charernsriwilaiwat N, Rojanarata T, Ngawhirunpat T, Opanasopit P (2014). Electrospun chitosan/polyvinyl alcohol nanofibre mats for wound healing. Int Wound J.

[24] Paipitak K, Pornpra T, Mongkontalang P, Techitdheer W, Pecharapa W (2011). Characterization of PVA-chitosan nanofibers prepared by electrospinning. Procedia Eng.

[25] Nokhasteh S, Molavi AM, Khorsand-Ghayeni M, Sadeghi-Avalshahr A (2019). Preparation of PVA/Chitosan samples by electrospinning and film casting methods and evaluating the effect of surface morphology on their antibacterial behavior. Materials Res Expr.

[26] Jiang D, Zhan H, Hu X, Luan J, Zhang M (2017). Preparation and performance of poly(d, L-lactic acid)-polyethylene glycol-poly(d, L-lactic acid) electrospun fibrous membranes for drug release. J Nanosci Nanotechnol.

[27] Hu J, Wei J, Liu W, Chen Y (2013). Preparation and characterization of electrospun PLGA/gelatin nanofibers as a drug delivery system by emulsion electrospinning. J Biomater Sci Polym Ed.

[28] Awan JA, Rehman SU, Bangash MK (2021). Development and characterization of electrospun curcumin-loaded antimicrobial nanofibrous membranes. Textile Res J.

[29] Rahalkar A, Kumathalli K, Kumar R (2021). Determination of efficacy of curcumin and Tulsi extracts as local drugs in periodontal pocket reduction: a clinical and microbiological study. J Indian Soc Periodontol.

[30] Agrawal A, Sharma AR, Rathod V (2024). Assessment of the efficiency of Tulsi extract as a locally administered medication agent and its comparison with curcumin in the treatment of periodontal pockets. Cureus.

